# *Orthosiphon aristatus* (Blume) Miq Alleviates Non-Alcoholic Fatty Liver Disease via Antioxidant Activities in C57BL/6 Obese Mice and Palmitic–Oleic Acid-Induced Steatosis in HepG2 Cells

**DOI:** 10.3390/ph16010109

**Published:** 2023-01-11

**Authors:** Salah Abdalrazak Alshehade, Raghdaa Hamdan Al Zarzour, Michael Mathai, Nelli Giribabu, Atefehalsadat Seyedan, Gurjeet Kaur, Fouad Saleih Resq Al-Suede, Amin Malik Shah Abdul Majid, Vikneswaran Murugaiyah, Hassan Almoustafa, Mohammed Abdullah Alshawsh

**Affiliations:** 1 Discipline of Pharmacology, School of Pharmaceutical Sciences, Universiti Sains Malaysia, Gelugor 11800, Penang, Malaysia; 2 Faculty of Bioeconomic & Health Sciences, Universiti Geomatika Malaysia, Kuala Lumpur 54200, Malaysia; 3 Department of Pharmacology, Faculty of Pharmacy, Arab International University, Damascus 16180, Syria; 4 College of Health and Biomedicine, Victoria University, Melbourne, VIC 3011, Australia; 5 Department of Physiology, Faculty of Medicine, Universiti Malaya, Kuala Lumpur 50603, Malaysia; 6 Department of Pharmacology, Faculty of Medicine, Universiti Malaya, Kuala Lumpur 50603, Malaysia; 7 Institute for Research in Molecular Medicine, Universiti Sains Malaysia, Gelugor 11800, Penang, Malaysia; 8 Eman Research Ltd., Level 3/81 Flushcombe Rd, Blacktown, NSW 2148, Australia; 9 Eman Biodiscoveries Sdn. Bhd., A1-4, Halal Park, Sungai Petani 08000, Kedah, Malaysia; 10 ACRF Department of Cancer Biology and Therapeutics, The John Curtin School of Medical Research, Australian National University, Canberra, ACT 2601, Australia; 11 Centre for Drug Research, Universiti Sains Malaysia, Gelugor 11800, Penang, Malaysia; 12 School of Clinical Sciences, Faculty of Medicine, Nursing and Health Sciences, Monash University, 246 Clayton Road, Clayton, VIC 3168, Australia

**Keywords:** *Orthosiphon aristatus*, non-alcoholic fatty liver disease, atherosclerosis, medicinal plant, high-fat diet, rosmarinic acid

## Abstract

Non-alcoholic fatty liver disease (NAFLD) is the most prevalent form of liver disease. *Orthosiphon aristatus* (Blume) Miq, a traditional plant in South Asia, has previously been shown to attenuate obesity and hyperglycaemic conditions. Eight weeks of feeding C57BL/6 mice with the standardized *O. aristatus* extract (400 mg/kg) inhibited the progression of NAFLD. Liver enzymes including alanine aminotransferase and aspartate transaminase were significantly reduced in treated mice by 74.2% ± 7.69 and 52.8% ± 7.83, respectively. Furthermore, the treated mice showed a reduction in serum levels of glucose (50% ± 5.71), insulin (70.2% ± 12.09), total cholesterol (27.5% ± 15.93), triglycerides (63.2% ± 16.5), low-density lipoprotein (62.5% ± 4.93) and atherogenic risk index relative to the negative control. Histologically, *O. aristatus* reversed hepatic fat accumulation and reduced NAFLD severity. Notably, our results showed the antioxidant activity of *O. aristatus* via increased superoxide dismutase activity and a reduction of hepatic malondialdehyde levels. In addition, the levels of serum pro-inflammatory mediators (IL-6 and TNFα) decreased, indicating anti-inflammatory activity. The aqueous, hydroethanolic and ethanolic fractions of *O. aristatus* extract significantly reduced intracellular fat accumulation in HepG2 cells that were treated with palmitic–oleic acid. Together, these findings suggest that antioxidant activities are the primary mechanism of action of *O. aristatus* underlying the anti-NAFLD effects.

## 1. Introduction

Non-alcoholic fatty liver disease (NAFLD) is the build-up of excess fat in the liver. Early-stage NAFLD does not usually present with symptoms but can progress to serious liver damage, including inflammation, called non-alcoholic steatohepatitis (NASH) and cirrhosis [1,2]. Due to the complex pathophysiology of NAFLD, it has recently been classified as a multi-systemic disease as it is usually associated with cardiovascular disease (CVD), chronic kidney disease, type 2 diabetes, obesity and dyslipidaemia [3].

Pathologically, oxidative stress is a key factor in the progression of NAFLD to advanced stages. On the other hand, prolonged abnormalities in lipid metabolism are closely associated with changes in oxidant/antioxidant balance, leading to cellular lipotoxicity, lipid peroxidation and chronic dysfunction of the endoplasmic reticulum and mitochondria [4].

Currently, NAFLD has a high prevalence (24%) worldwide [5], which has resulted in the redirecting of resources and research funds to support studies attempting to identify the high-risk NAFLD population. Additional studies are focusing on understanding the mechanism of progression and, in turn, identifying potential molecular targets for treatment. To date, there is no approved anti-NAFLD drug, and the treatment options are based on prophylactic strategies, lifestyle modifications and physical activity [6]. Several emerging potential therapies based on antioxidant effects have increasingly been studied and developed. Considering the multiple mechanisms underlying the lipid metabolism of medicinal plants with potent bioactive composition, they could act on multiple arms of disease pathophysiology [7].

*Orthosiphon aristatus* (Blume) Miq. is a medicinal plant widely used in Southeast Asian countries such as Malaysia and Indonesia, and traditionally used to treat cystitis, diabetes, rheumatism, arthritis, gout and kidney disease [8]. This broad spectrum of benefits is attributed to the rich phenolic content, including polyphenols, diterpenes, triterpenes, lipophilic flavones, glycosides and caffeic acid derivatives such as rosmarinic acid (RA) [9,10]. *O. aristatus* is reported to have α-amylase and β-glucosidase inhibitory activities, exhibits antioxidant and anti-inflammatory properties, regulates lipid metabolism, promotes insulin secretion, improves insulin resistance, increases glucose uptake, promotes glycolysis and inhibits gluconeogenesis [11]. More importantly, a previous study provides evidence for the anti-obesity effect of *O. aristatus* through the inhibition of pancreatic lipase [12]. Thus, *O. aristatus* is suggested to exhibit anti-NAFLD effects through inhibiting fat accumulation, antioxidant effects and its potential antidiabetic activities. In the present study, the amelioration of *O. aristatus* ethanolic extract and its fractions on NAFLD has been investigated using C57BL/6 mice fed with a high-fat diet (HFD) and palmitic–oleic acid-induced HepG2 cell line.

## 2. Results

### 2.1. O. aristatus Standardization

**Figure 1:**
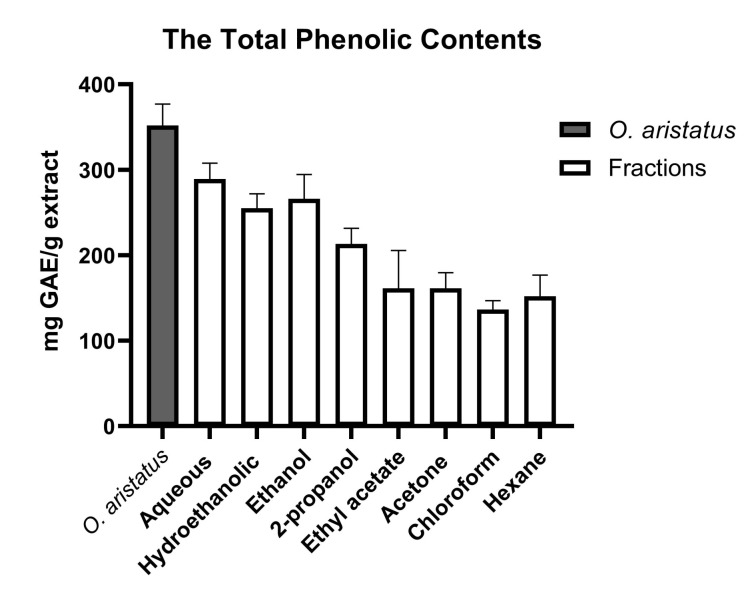
Total phenolic contents of *O. aristatus* extract and its fractions. Values were presented as mean ± SD, *n* = 3.

As shown in Figure 1, the polar fractions of *O. aristatus* (ethanolic, hydroethanolic and water) had a higher phenol content compared to the non-polar fractions (hexane, chloroform and acetone). The aqueous fraction had the highest phenol content (289.3 ± 18.5 mg GAE/g extract), followed by the hydroethanolic (ethanolic 50%) and ethanolic 100%, which have almost similar values (255.0 ± 17.1 and 266.0 ± 28.6 mg GAE/g extract, respectively). Additionally, the chloroform fraction (136.5 ± 10.4 mg GAE/g extract) shows the lowest phenol concentration.

**Figure 2:**
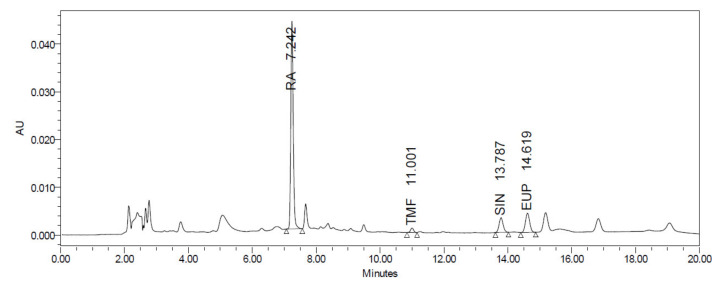
HPLC chromatogram of the *O. aristatus* extract analysis.

The HPLC analysis revealed that *O. aristatus* contained several peaks at different retention times (Figure 2), demonstrating that multiple components were detected. Rosmarinic acid (RA) was the major compound with the highest peak appearing at 7.242 min with a corresponding content of 61.96 ± 0.009 µg/mg extract (6.1%) (Table 1). Further, 3′-hydroxy-5,6,7,4′-tetramethoxyflavone had the lowest content of 0.72 ± 0.005 µg/mg extract.

### 2.2. In-Vivo Results

#### 2.2.1. Effects of *O. aristatus* on Body Weight, Organ Weight and Food Intake

**Figure 3:**
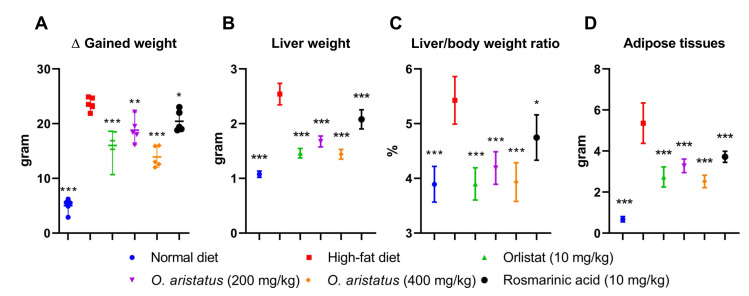
The effects of *O. aristatus* on NAFLD-related mice organs. (**A**), the difference in body weight between stat and end tome point of the experiment. (**B**), the liver weight at the end of the experiment. (**C**), the ratio of the liver weight to the final body weight. (**D**), the total weight of the adipose tissue. * *p* < 0.05, ** *p* < 0.01, *** *p* ≤ 0.001 compared to high-fat diet control group. The values were presented as mean ± SD, *n* = 5.

At the end of the treatment, the HFD group showed a significant gain in body weight, liver and adipose tissue weight compared to the ND group (*p* < 0.001) (Figure 3A,B,D, respectively). The treated groups gained more than 50% compared to the ND (Figure 3A); however, mice treated with orlistat (10 mg/kg) and *O. aristatus* (400 mg/kg) showed a significant reduction in the gained weight compared to the untreated HFD group. Further, the liver weight showed a significant reduction in all the groups compared to the HFD group (Figure 3B), which is also reflected in a similar pattern in the liver-to-body weight ratio (Figure 3C). Further, treating mice with a high dose of *O. aristatus* (400 mg/kg) for two months significantly reduced the abdominal adipose tissue weight (*p* < 0.001) as compared to the HFD group (Figure 3D).

#### 2.2.2. Effects of *O. aristatus* on Serum-Related Parameters

**Figure 4:**
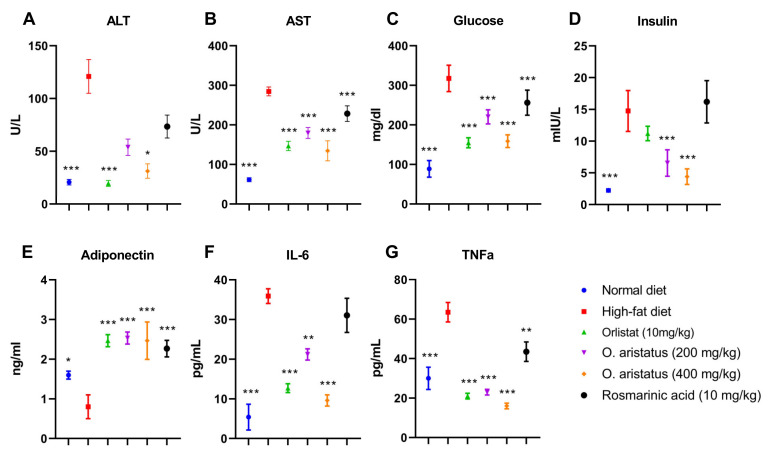
The effects of *O. aristatus* on liver enzymes, serum glucose, serum insulin, adiponectin and inflammatory cytokines. * *p* < 0.05, ** *p* < 0.01, *** *p* ≤ 0.001 compared to high-fat diet control group. The values were presented as mean ± SD, *n* = 5.

**Figure 5:**
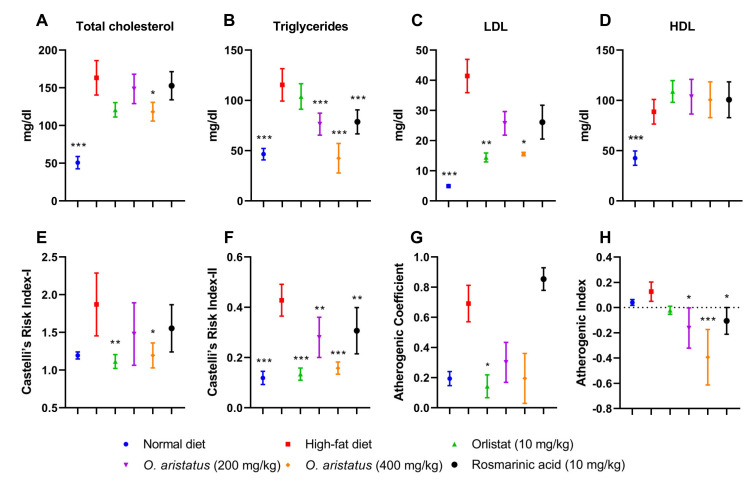
The effects of *O. aristatus* on serum lipid profile and the atherogenic lipids ratios. * *p* < 0.05, ** *p* < 0.01, *** *p* ≤ 0.001 compared to the high-fat diet group. Values were presented as mean ± SD, *n* = 5.

Feeding HFD for four months significantly elevated the serum concentrations of ALT, AST, glucose, insulin, triglyceride (TG), cholesterol, and LDL as compared to the ND group (*p* < 0.001) (Figure 4 and Figure 5). Comparatively, the ALT concentration was significantly reduced by treatment with a high dose of *O. aristatus* (400 mg/kg) when compared to the HFD group (*p* < 0.05) but was not in the case of mice treated with a low dose (200 mg/kg) or the RA (Figure 4A). Further, AST levels were significantly reduced in all treated groups compared to the HFD group (Figure 4B). A similar pattern was noticed for the glucose concentration (Figure 4C).

Moreover, as shown in Figure 4D, the level of mice serum insulin concentration at the end of the treatment was significantly reduced in mice treated with orlistat and both doses of *O. aristatus*, but not in the RA group when compared to the HFD group. Further, the Pearson correlation test (Table 2) indicated that the insulin and glucose levels were positively correlated in HFD, *O. aristatus* (200 mg/kg), and RA groups, while the correlation was negative in ND, orlistat and *O. aristatus* (400 mg/kg) groups.

Similarly, HOMA-IR indicated the ND group is the only group that showed insulin sensitivity, and treating mice with *O. aristatus* (400 mg/kg) recovered the insulin resistance condition that was shown in the HFD group (Table 3). However, treatment with orlistat and *O. aristatus* (200 mg/kg) for two months reduced the insulin resistance condition, and a similar pattern was indicated with the quantitative insulin-sensitivity check index (QUICKI) (Table 3).

Further, serum adiponectin levels were significantly lower in the HFD-fed mice compared to the ND-fed mice, in contrast with all the treated groups which showed higher levels of adiponectin compared to both HFD and ND groups (Figure 4E). Along with increased body weight and insulin-resistance conditions, serum proinflammatory cytokines, including IL-6 and TNF-α, also showed a significant increase in comparison with the ND group, which were reversed after treating mice with orlistat and both doses of *O. aristatus* for two months (Figure 4F,G).

The serum lipid profile showed disparate results; a significant reduction in the cholesterol level was noticed only in mice treated with *O. aristatus* (400 mg/kg) when compared with the HFD group (Figure 5A). Further, the two doses of *O. aristatus* significantly reduced TG serum levels but were not reduced in the positive control (orlistat) group when compared with the HFD group (Figure 5B). Conversely, orlistat significantly reduced the concentration of LDL with similar efficacy as the high dose of *O. aristatus* (Figure 5C). Interestingly, neither of the treatment groups showed a significant elevation of HDL serum concentration when compared to the HFD group (Figure 5D). Furthermore, as dyslipidaemia is the main risk factor for CVD, we calculated the lipids ratio to predict the anti-atherogenic effect of *O. aristatus* extract (Figure 5E–H). Results demonstrated that the high dose of *O. aristatus* (400 mg/kg) and the positive control (orlistat) was significantly able to ameliorate the atherogenic lipids ratios.

### 2.3. Histological Assessment

**Figure 6:**
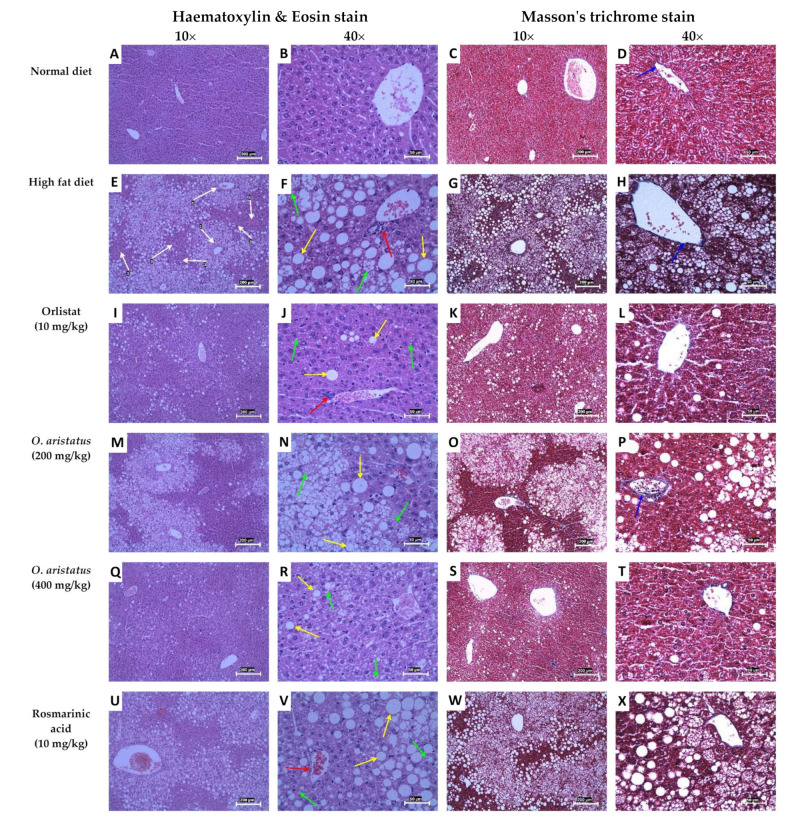
Hematoxylin and eosin and Masson’s trichrome staining of mice liver tissues. The yellow arrows indicate macrovesicular steatosis within hepatocytes, the green arrows indicate microvesicular steatosis, the red arrows indicate lobular inflammation and the blue arrows indicate the blue-stained areas that are the connective tissue of the portal tracts. The white arrows indicate the liver tissue zones, zone 1 encloses the portal vein (hepatic arteries), zone 3 lies around central veins and zone 2 lies in between. The 10× scale bar is 200 µm and the 40× scale bar is 50 µm.

The definitive diagnosis of NAFLD steatosis is made in a liver biopsy, as the fat accumulates in the hepatocytes as vacuoles, which give a clear appearance with H&E staining (Figure 6). Microscopically, the normal liver parenchyma with portal tracts without any fat accumulation can be observed clearly in Figure 6A–D. Feeding mice with HFD for 16 weeks caused remarkable hepatic lipid accumulation with slight inflammation (Figure 6E–H and Table 4) compared to the ND group. The fat accumulated around the portal tracts (zone 1), especially microvesicular steatosis, and near zone 2 macrovesicular accumulation was seen (Figure 6G). The fat accumulation markedly decreased after eight weeks of orlistat and the high dose of *O. aristatus* treatment, represented in the NAS scoring result (Table 4) and microscopically in Figure 6I,J,Q,R. Although the lobular inflammation was minimal in HFD, low and high doses of *O. aristatus* showed a reduction in inflammation levels (Table 4). Interestingly, the mice treated with RA showed no reduction in steatosis and had a similar pattern as the negative control (Figure 6U–X). Masson’s trichrome staining revealed collagen fibres normally present in the portal tracts with no evidence of fibrosis (Figure 6D,H,L,P,T,X).

### 2.4. Antioxidant Enzymes Activity in the Liver Tissue

**Figure 7:**
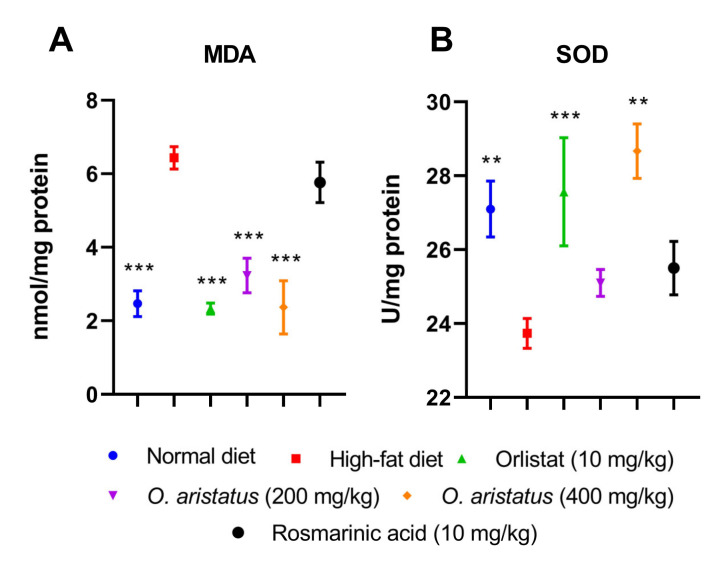
The effect of *O. aristatus* on the malondialdehyde (MDA) and superoxide dismutase (SOD). * *p* < 0.05, ** *p* < 0.01, *** *p* ≤ 0.001 compared to high-fat diet group. Values were presented as mean ± SD, *n* = 5.

As shown in Figure 7, treating the mice with a high-dose of *O. aristatus* and orlistat for 8 weeks significantly increased (*p* < 0.01) the SOD levels and alleviated the lipid peroxidation, indicated by the significant reduction in malondialdehyde (MDA) compared to the HFD group. Similarly, the low-dose administration of *O. aristatus* significantly reduced the MDA level (*p* < 0.001).

### 2.5. In Vitro Results

#### 2.5.1. Cytotoxicity Assay

**Figure 8:**
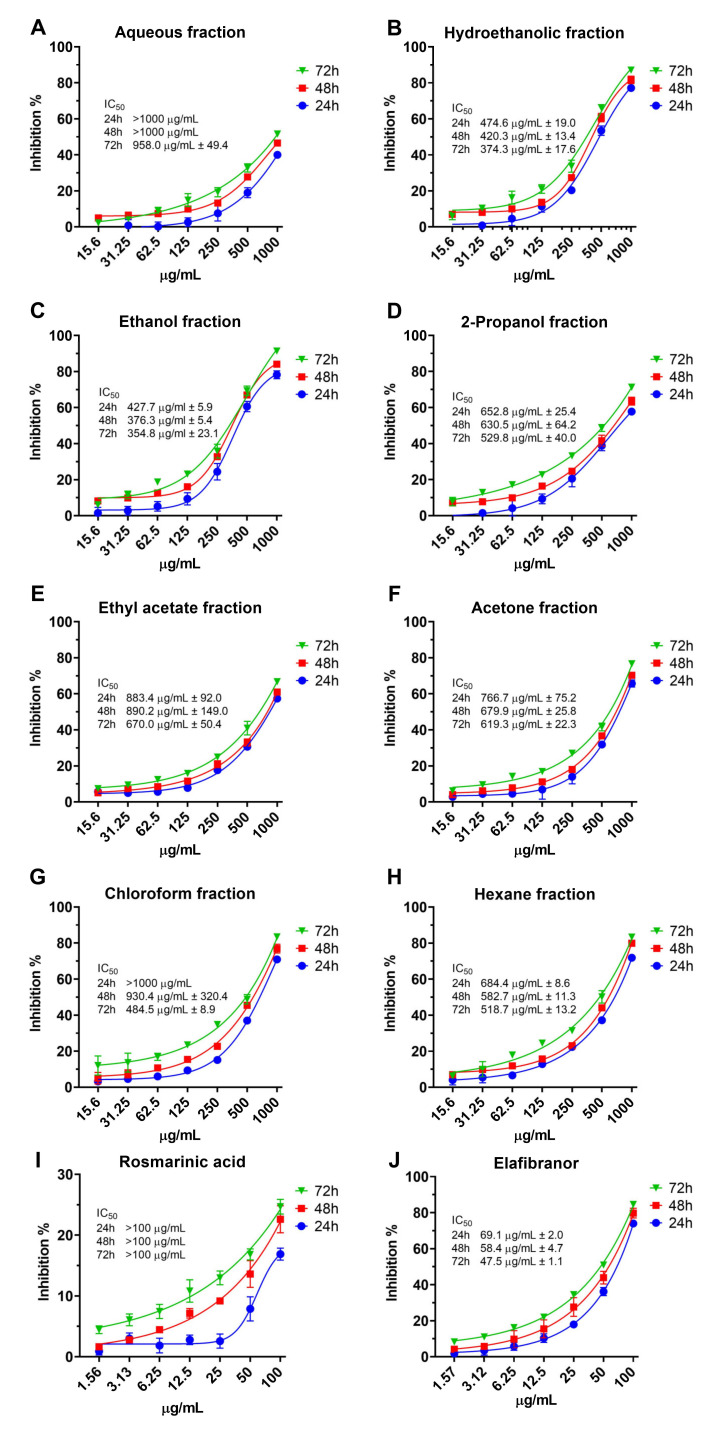
The cytotoxicity of *O. aristatus* fractions against HepG2 cells. Concentration scales were logarithmized to obtain relative response curves. Values were presented as mean ± SD, *n* = 3.

The result of the cytotoxicity assay (Figure 8A–J) showed that the aqueous fraction has relatively the lowest toxic effect (IC_50_ > 1000 µg/mL after 24h and 48h) compared to the other fractions, even after the 72 h of treatment the IC_50_ was 958.0 ± 49.4 µg/mL. After 24 h, the hydroethanolic and ethanolic fractions had the lowest IC_50_ (374.3 ± 17.6 µg/mL and 354.8 ± 23.1 µg/mL, respectively). The RA showed relatively low cytotoxicity (Figure 8I) with IC_50_ > 100 µg/mL. Furthermore, we tested the cytotoxicity of elafibranor as a positive control on the same cell line as indicated in Figure 8J; elafibranor showed high potency in inhibiting cell growth with an IC_50_ value of 48.6 ± 1.451 µg/mL. Moreover, all fractions’ concentrations of 150 µg/mL and below were not cytotoxic and the percentage of inhibition was below 20%, therefore 150, 75 and 35 µg/mL were chosen for further assays.

#### 2.5.2. Intracellular Fat Accumulation

**Figure 9:**
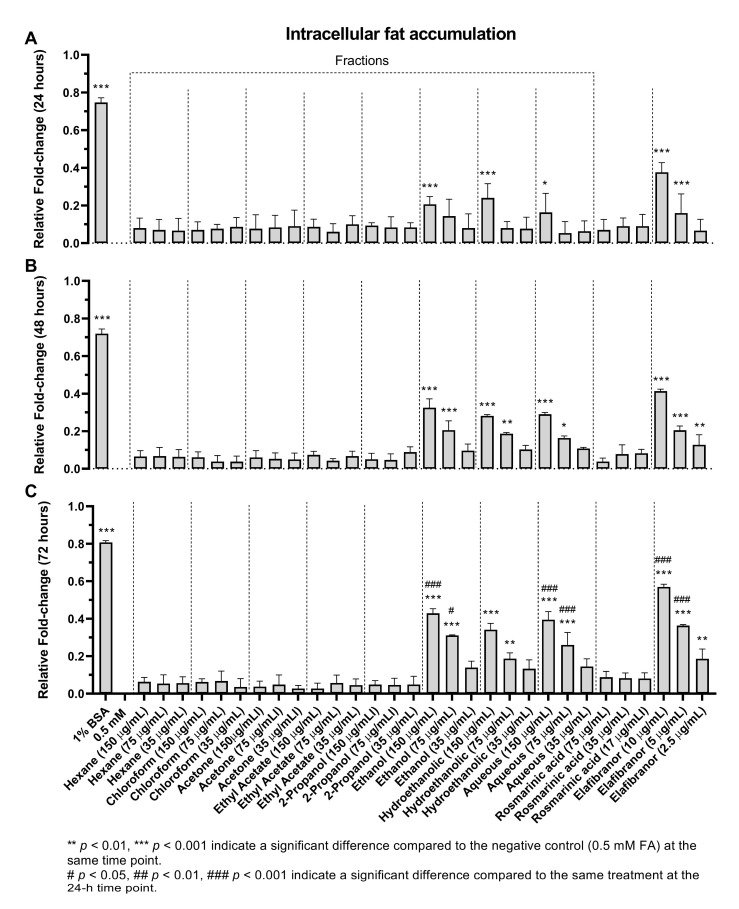
The effects of *O. aristatus* fractions on the intracellular fat accumulation. Fat accumulation level was tested after treating the steatotic cells with different concentrations of *O. aristatus* fractions at different time points (24 h (**A**), 48 h (**B**), and 72 h (**C**)). The fat content was normalized per cell and then presented as fold change compared to 0.5 mM FA as a negative control. Values were presented as mean ± SD, *n* = 3, * *p* is 0.05.

**Figure 10:**
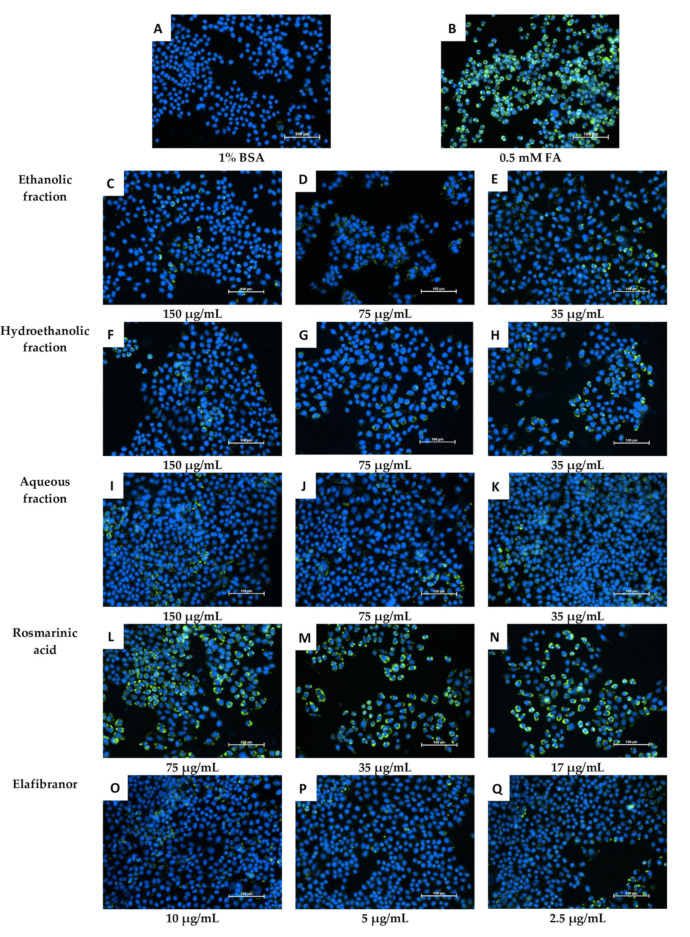
Microscopic examination of the effect of *O. aristatus* fractions on the intracellular lipid accumulation (24 h). The green colour is the Nile Red stain (FITC filter) indicating the fat droplet inside the cells and the blue colour is Hoechst 33342 (DAPI filter) as a nuclear counterstain. The scale bar is 100 µm (20×).

Based on the data obtained (Figure 9), it can be suggested that the non-polar fractions (hexane, chloroform, acetone, ethyl acetate and 2-propanol) did not reduce the intracellular lipid accumulation in the HepG2 cells (Figure 9 and Figure 10) even after 72 h of treatment (Figure 9C). However, the polar fractions (ethanol, hydroethanolic and aqueous) showed a significant reduction in intracellular fat accumulation compared to the negative control. The effectiveness of the different polar and non-polar fractions can be observed in the fluorescence microscopic examination results (Figure 10). In addition, after 24 h, the highest concentration (150 µg/mL) of ethanolic fraction and hydroethanolic showed a significant reduction in intracellular fat content (21% and 24%, respectively) compared to 0.5 mM FA. However, after 24h, the aqueous fraction reduced the fat content by 17%, which became significant after 48h of treatment compared to 0.5 mM FA (29%, *p* < 0.005) (Figure 9A,B). Furthermore, the positive control (elafibranor) revealed a significant reduction in intracellular fat by 34% compared to the negative control (*p* < 0.001) (Figure 9A). Furthermore, RA showed no significant activity over the three-time points, and none of the treatments showed a significant difference between the 24-h and 48-h duration (Figure 9B). Therefore, treatment for 24 h was chosen for further in vitro investigations, and the polar fractions (ethanol, hydroethanolic and aqueous) were chosen for the downstream assays because of their remarkable effect.

### 2.6. Antioxidant Activities

**Figure 11:**
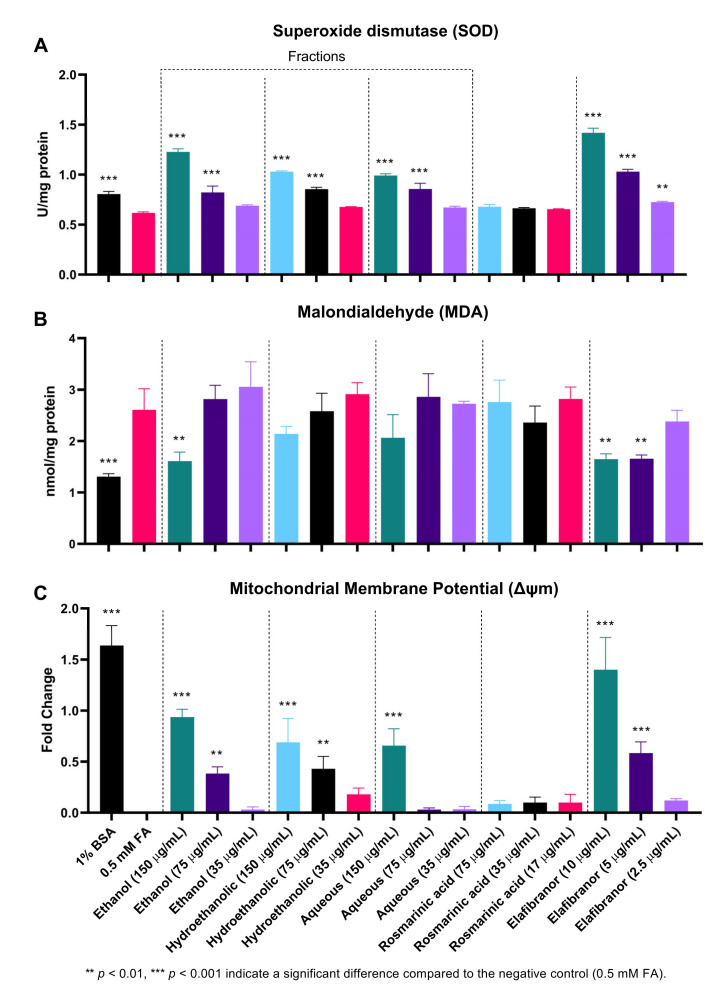
The effect of *O. aristatus* fractions on the antioxidant enzyme superoxide dismutase (SOD) (**A**), Malondialdehyde (MDA) (**B**), and the mitochondrial membrane potential (MMP) (**C**). Values were presented as mean ± SD, *n* = 3.

As indicated in Figure 11A, SOD activity was significantly decreased in the negative control (0.5 mM FA) compared to the uninduced group (1% BSA). Moreover, it can be noted that the high and low concentrations (150 µg/mL and 75 µg/mL) of all tested fractions showed a significant increase in SOD levels, which suggests an increase in the antioxidant capacity of the cells. Furthermore, treatment of the cells with different concentrations of RA for 24 h showed no significant difference compared to the control group (0.5 mM FA).

Throughout the course of this experiment, the MDA level was only significantly reduced in the high dose of the ethanolic fraction compared to the control (0.5 mM FA). The similar concentration of the other two fractions showed a reduction but was not statistically significant. However, RA had no significant effect on reducing MDA levels, in contrast to the positive control (elafibranor) which was found to significantly reduce MDA levels at two concentrations, 10 µg/mL and 5 µg/mL (Figure 11B).

**Figure 12:**
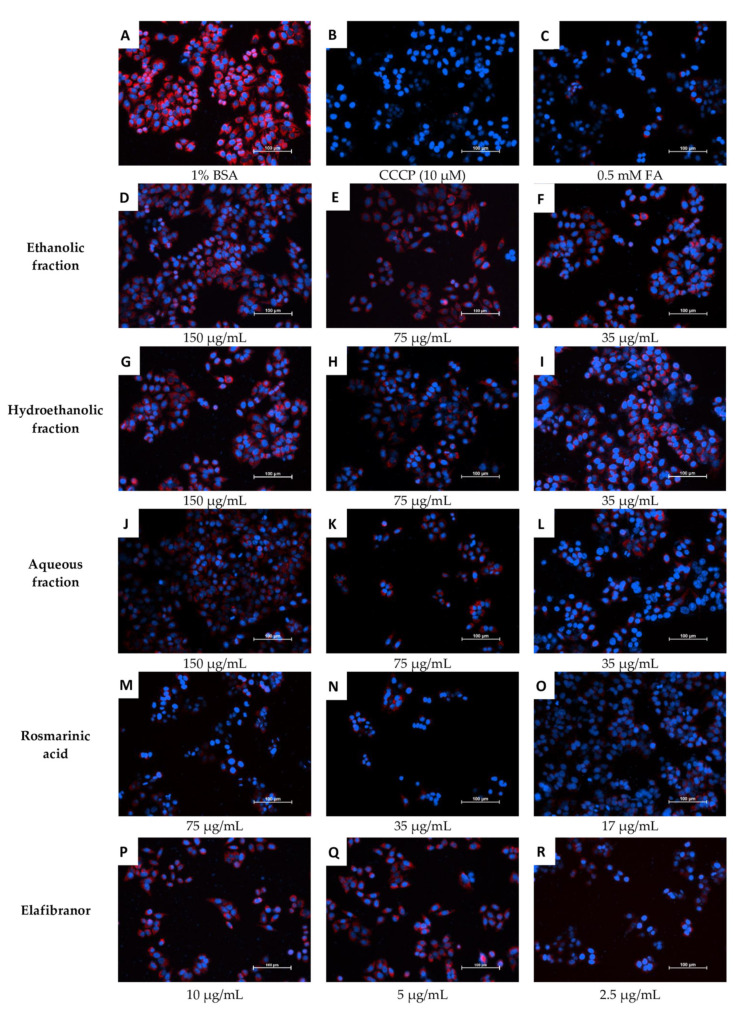
Microscopic examination of the effects of *O. aristatus* fractions on the mitochondrial membrane potential. The red colour is the TMRM stain (TRITC filter), and the blue is Hoechst 33342 (DAPI filter) as a nuclear counterstain. BSA: Bovine Serum Albumin, CCCP: Carbonyl Cyanide Chlorophenylhydrazone, FA: Fatty Acids Mixture. The scale bar is 100 µm (20×).

Figure 11C and Figure 12 showed that the ethanolic, hydroethanolic and aqueous fractions significantly restored the mitochondrial membrane potential (MMP), thereby maintaining the oxidation process. This pattern is correlated and consistent across inhibitory effects against intracellular fat accumulation.

## 3. Discussion

Despite the growing understanding of the pathophysiology of NAFLD, lifestyle modification with regular exercise and dietary changes are the only proposed management approach. To date, there is no approved treatment available for NAFLD. Medicinal herbs with high phenolic content have been explored as a safe and convenient treatment option for NAFLD, as they are known to have beneficial anti-inflammatory and antifibrogenic activity, and antioxidant effects, and can suppress hepatic lipid accumulation [17]. The current study showed strong evidence of the anti-steatotic effect of *O. aristatus* by using HFD-fed mice and a palmitic–oleic acid-induced model of NAFLD in HepG2 cells. Our standardized *O. aristatus* extract not only suppressed hepatic fat accumulation but also showed significantly lower risk indicators of atherosclerosis compared to untreated mice.

Starting with the in vivo outcomes, feeding C57BL/6 mice with a 60% fat diet for two months successfully developed NAFLD. This was initially noticeable by a 1.5× increase in liver weight and 4× in adipose tissue compared to the ND group. The high increase in the weight of the liver and visceral fat, aside from the development of hyperlipidemia in the HFD group, is a prominent feature of NAFLD [18,19,20]. Typically, NAFLD is characterized by mild elevations (one- to two-times the upper limit of normal) in serum ALT and AST levels in addition to the abnormal hepatic content of SOD and MDA; these characteristics in HFD-fed mice are strong indicators of liver damage and oxidative stress [21,22]. Moreover, ALT and AST both elevated four-fold compared to the ND group, which confirms the hepatic injury in the HFD group. These conditions also led to an insulin resistance state, which was observed by the increased levels of fasting serum glucose and insulin, and was confirmed by HOMA-IR and QUICKI. The current results are consistent with previous studies and further support the ability of HFD to induce accumulation of fat in the liver with marked increases in insulin resistance conditions [23], which in turn increases oxidative stress in the liver causing hepatic lipid peroxidation and triggers inflammatory responses [24]. Moreover, an insulin resistance condition is usually associated with adipose tissue dysfunction and ectopic lipid deposition [2,25].

Furthermore, as expected, a higher risk of atherosclerosis was noticed among the HFD group, confirmed by the increase in serum atherogenic indexes, which was attributed to the sharp evaluation in the serum lipid profile including total cholesterol, TG and LDL when compared to the ND group. The overload of LDL is usually oxidized to form peroxides, which play a role in several atherosclerotic stages through their cytotoxic effects, leading to endothelial injury [22]. This confirms the positive correlation between atherosclerosis and NAFLD [26].

More importantly, histological evaluation, which is the gold standard in NAFLD diagnosis demonstrated that feeding mice with HFD for 16 weeks resulted in a clear manifestation of NAFLD i.e., micro-, and macro-vesicular steatosis with minimal foci of inflammation, confirming the development of steatosis (NAFLD) but not NASH. Nonetheless, serum levels of the proinflammatory markers (i.e., IL-6 and TNFα) were significantly higher in the HFD group compared to the ND group. The severity of NAFLD is associated with an increase in oxidative stress and proinflammatory status [27]. Furthermore, the serum levels of adiponectin were significantly reduced by feeding mice with HFD for four months when compared to the ND group. Adiponectin has been confirmed to primarily target the liver, particularly in fatty liver disease, as it can control many liver functions, including metabolism, inflammation and fibrosis [28].

The significant total phenolic content of the *O. aristatus* extract was in line with the previous studies [29]. The outcomes showed that the polar fractions have a higher phenolic content compared to the non-polar fractions, the aqueous fraction of *O. aristatus* contained the highest phenol content among all extracts, followed by the ethanolic fraction. It was revealed that polar solvents effectively dissolved the phenolic compounds which have hydroxyl groups that make them more hydrophilic [30], and also function as hydrogen donors, thus reflecting good antioxidant activity [31]. Furthermore, the concentration of the four markers (i.e., sinensetin, eupatorin, 3′-hydroxy-5,6,7,4′-tetramethoxyflavone, and RA) was in line with previous studies [31,32].

An eight-week feeding of *O. aristatus* standardized extract showed a significant reversal of NAFLD status in mice. Parameters such as body weight, liver weight and liver enzymes (ALT and AST) showed a significant reduction compared to the HFD group. Notably, adiponectin levels were restored after treatment with *O. aristatus*. These results support previously reported findings of a hepatoprotective effect of *O. aristatus* [33]. A previous study reported reduced serum levels of ALT and AST in addition to hepatic necrosis [34]. Another study conducted in a diabetic rat model evaluating the antidiabetic effects of *O. aristatus* aqueous extract showed that there was a significant reduction in blood glucose levels [29]. The hepatoprotective effects of *O. aristatus* have been attributed to its antioxidant activity, free radical scavenging properties and the ability to prevent the depletion of tissue glutathione levels [10,34,35].

In addition, liver functions were improved by *O. aristatus*, as indicated by a reduction in the serum levels of glucose, insulin and HOMA-IR, indicating a significant decrease in insulin resistance. HOMA-IR is an important indicator of insulin resistance. It was found that being overweight or obese was persistently associated with higher HOMA-IR [36]. A HOMA-IR of 4.5 was estimated to be an optimal threshold for discriminating NAFLD from non-NAFLD cases [15]. In this study, the HOMA-IR in the negative control was 11.69 ± 3.247, which is an additional confirmation of the development of NAFLD. On the other hand, the high dose of *O. aristatus* was able to reduce the HOMA-IR value to 1.70 ± 0.463, indicating a significant reverse in the insulin resistance condition. This result is in line with several previous studies that have proposed *O. aristatus* as an alternative treatment for type 2 diabetes [11,29,37].

Furthermore, the results of this study suggested that *O. aristatus* improved crosstalk between the liver and adipose tissue, as indicated by significantly improved adiponectin, an adipokine secreted by adipocytes, levels after treatment with high and low *O. aristatus* doses for two months. Adiponectin is a well-known homeostatic factor for regulating glucose levels, lipid metabolism and insulin sensitivity through its anti-inflammatory, anti-fibrotic and antioxidant effects [38,39]. This was reflected in the reduction of the serum lipid profiles (i.e., TG. TC and LDL) compared to the negative control group (HFD), particularly with the high dose (400 mg/kg) of *O. aristatus*. This provides insight and demonstrates the beneficial usage of *O. aristatus* to protect against dyslipidaemia and CVD, as the abnormalities in lipid profile values are directly associated with susceptibility to atherosclerotic complications [40]. The atherogenic lipid ratios are validated as powerful predictors that can be used as a standalone index to estimate CVD [41,42].

These observations were also supported by findings from the histological assessment, where H&E staining showed a marked reduction in accumulated fat and inflammation after *O. aristatus* (400 mg/kg) treatment for 8 weeks. One possible mechanism of this beneficial effect is the powerful antioxidant effects that could result from enhancing the SOD levels, which are ideally higher than the negative control in both mice and HepG2 cell NAFLD-induced models, and reducing oxidative levels reflected by low MDA to similar levels compared to the ND group. More specifically, the accumulated fats in the liver induce the release of ROS associated with lipid peroxidation markers such as MDA [22], which in turn is considered an indicator of decreased antioxidant activity, especially in a patient with NAFLD [43]; a similar pattern was noticed with antioxidant enzymes such as SOD [44].

This study was further carried out with a series of in vitro experiments to examine the most active fraction of *O. aristatus*. The polar and non-polar fractions were revealed to have relatively low cytotoxicity against the HepG2 cell line and the aqueous fraction showed the lowest cytotoxicity with an IC_50_ > 1000 µg/mL which is consistent with a previous study result [45]. A recent study found that the IC_50_ of the RA against HepG2 cells was 270.14 ± 73.33 µg/mL, which matches our results (>100 µg/mL). However, they found that the ethyl acetate fraction has a relatively lower IC_50_ (130.82 ± 17.64 µg/mL) against HepG2 [46]. This deviation could be due to different extraction methods, thus different concentrations of the fraction composition [10,47]. Overall, *O. aristatus* is considered a safe plant, as it was tested for acute toxicity in a rat model with LD50 of more than 5000 mg/kg [48].

After confirming the low cytotoxicity of the fractions, three concentrations were chosen to investigate the intracellular fat accumulation directly in the cells using a fluorometer and compared to fatty acids mixture (i.e., oleic and palmitic acids), it was reported that an increased uptake of oleic and elaidic acids resulted in altered expression of fat metabolism genes [49]. It was surprising that only the more polar fractions (ethanolic, hydroethanolic and water) had effects, particularly after 24 h of treatment. Although the hydroethanolic fraction with the highest concentration (150 µg/mL) had the most potent effect in reducing the intracellular fat accumulation at 24 h, the ethanolic fraction showed a significant effect even at 75 µg/mL concentration after 48 and 72 h. These results suggest that it is more likely the ethanolic fraction has a more potent effect, especially at longer duration treatment. Similar to the in vivo results, the RA also showed no anti-fat accumulation, despite being the main compound of the extract, suggesting that the anti-NAFLD effects of *O. aristatus* could be attributed synergistic effects of different bioactive compounds in this plant.

The initial assumptions regarding the correlation of the antioxidant activity of the *O. aristatus* fractions with anti-NAFLD activity are valid. The polar fractions, showing reduced intracellular fat accumulation, also showed increased SOD enzyme activity similar to the in vivo findings. However, the ethanolic fraction showed the highest effects, which appear more clearly in the MDA measurement.

Furthermore, since alterations in mitochondria can cause NAFLD, we examined the mitochondrial membrane potential as it is the central intermediate in oxidative energy metabolism and indicates the functional work of the mitochondria [50]. The highest concentration of all selected fractions showed a significant restoration of mitochondrial activity, which is supporting evidence for the antioxidant-based anti-NAFLD mechanism, especially when it correlates with high total phenolic content levels of the fractions recorded in this study and is consistent with the previous study results [51,52]. It has been established that phenolic compounds inhibit the radical processes of oxidation of substrates through the action of semiquinone present in the equilibrium system [53]. This is in agreement with the available data that reported the high radical scavenging ability of aqueous, ethanolic and methanolic extracts of *O. aristatus* using the 2,20-diphenyl-1-picrylhydrazyl method [54].

Free radicals such as ROS, including hydroxyl radicals, superoxide anions and hydrogen peroxide, play an important role in the progressive destruction of living tissue; they are also responsible for many metabolic diseases such as NAFLD, obesity, atherosclerosis, heart disease, old age and cancer [55]. Within the cells, ROS are mainly produced in the mitochondria, the peroxisomes and the ER, but there is also cytoplasmic production of ROS. High levels of ROS can alter organelles, further increasing oxidative stress and creating an ongoing cycle [55]. Additionally, taking into consideration that the ethanolic fraction has a moderate relative polarity of 0.654 [56], suggesting that it can dissolve polar and non-polar compounds, leads to the conclusion that the ethanolic fraction contains the most bioactive compounds of *O. aristatus*.

In addition, this work is also limited by not considering the lipogenesis and lipolysis effects of *O. aristatus*. As an extension of the current work, it would be useful to use human multilineage 3D spheroids or organoids as a model to illustrate the molecular mechanisms of the anti-NAFLD effects of *O. aristatus* extract and its polar fractions. It would also be of interest to elucidate the beneficial effect of formulating *O. aristatus* with other medicinal herbal extracts or even antidiabetic and obesity treatment to enhance the anti-NAFLD efficacy.

## 4. Materials and Methods

### 4.1. Plant Extraction Preparation

The fresh leaves of *O. aristatus* were collected from the Universiti Malaya Botanical Garden and then identified by the KLU herbarium (code: KLU49065). The leaves were dried in an oven (40 °C) and ground to a coarse powder. Subsequently, 400 g of powdered *O. aristatus* leaves were extracted by the maceration method using 4 L of 95% ethanol (R&M Chemical, Milton Park, United Kingdom, Cat. 64-17) at room temperature with occasional shaking for two days [57]. The *O. aristatus* extract was filtered using Whatman grade 1 filter paper, concentrated using a vacuumed rotary evaporator (Rotavapor R-200, Buchi, Switzerland), and stored in a freezer at −20 °C.

### 4.2. Fractionation by Column Chromatography

The stationary phase of column chromatography was prepared by mixing 50 g of silica gel 60 H powder (Merck, Germany, Cat. 1.07736) with 300 mL hexane, then immediately transferred into a glass column (22 × 400 mm) and washed three times with hexane. After that, 500 mg of extract of *O. aristatus* was dissolved in 2.5 mL of methanol (100%) and loaded on the top of the stationary phase. The column was eluted stepwise with 25 mL of each of the fractionation solvents according to the polarity, including hexane, chloroform, acetone, ethyl acetate, 2-propanol, ethanol, ethanol:water (1:1) and water. The flowed-down solvent was reloaded three times before moving on to the next solvent. Finally, the fractions were collected inside labelled glass tubes with screw covers, and the vacuum centrifuge was used for two hours to evaporate the solvents.

### 4.3. Standardization of the Extract

#### 4.3.1. Total Phenolic Content

The total phenolic content of the extract and the fractions was determined spectrophotometrically using the Folin–Ciocalteu assay [58]. Briefly, the Folin–Ciocalteu reagent was diluted with dH_2_O in a ratio of 1:10. Gallic acid was prepared as a standard with different concentrations (i.e., 0, 10, 50, 100, 150, 200 µg/mL). Then, serial dilutions of quercetin (positive control) and each *O. aristatus* fraction were prepared at 0, 25, 50 and 100 µg/mL. In a microcentrifuge, 10 µL of the standard, each fraction, extract and positive control were mixed with 500 µL Folin–Ciocalteu reagent and incubated for 5 min. Then, 350 µL of sodium carbonate (115 mg/mL) was added, mixed and incubated for 2 h before reading absorbance at 765 nm. The total phenolic content was expressed as mg gallic acid equivalent (mg GAE) per gram of the extract/fraction.

#### 4.3.2. High-Performance Liquid Chromatography (HPLC)

The reverse-phased HPLC analysis method was adapted from previous studies [31,32,59,60]. Briefly, a filtered stock solution (5 mg/mL) of *O. aristatus* extract was prepared in methanol: water (1:1). Similarly, all reference compounds including 3′-hydroxy-5,6,7,4′-tetramethoxyflavone, sinensetin, eupatorin and RA was prepared in a stock of 1 mg/mL, then a serial dilution was prepared (100, 50, 25, 12.5, 6.25, 3.13, 1.56, 0.78 µg/mL). The chromatographic separation was carried out using a gradient mobile phase 0.1% formic acid: acetonitrile at a flow rate of 1 mL/min on a C18 column (3 µm, 3 × 150 mm) and HPLC device equipped with a diode array detector (1260 Infinity II LC System, Agilent, Santa Clara, CA, USA). The entire analysis time was 20 min and absorbed at a λmax of 320 nm.

### 4.4. In Vivo Experiments

#### 4.4.1. Animal Study

Seven-week-old wild-type male black mice (C57BL/6; ≈20 g) were purchased from the Animal Experimental Unit (AEU), Faculty of Medicine, Universiti Malaya. Mice were housed under standard laboratory conditions (≈25 °C; ≈75% humidity, a 12-h dark–light cycle) for one week. Mice had access to food and water *ad libitum*. The experiment was conducted following the National Research Council’s Guide for the Care and Use of Laboratory Animals with ethical approval number USM/IACUC/943/114 [61].

Thirty mice were randomly divided into six groups as follows: normal control group with normal diet (ND), negative control group with HFD (60% Kcal fat, ENVIGO, Indianapolis, IN, USA, Cat. TD.06414), positive control group with HFD and 10 mg/kg orlistat (Cayman, Ann Arbor, MI, USA, Cat. 10005426), low dose treatment group with HFD and *O. aristatus* (200 mg/kg), high dose treatment group with HFD and *O. aristatus* (400 mg/kg), last treatment group with HFD and 10 mg/kg RA (Sigma-Aldrich, Saint Louis, MO, USA, Cat. R4033).

**Figure 13:**
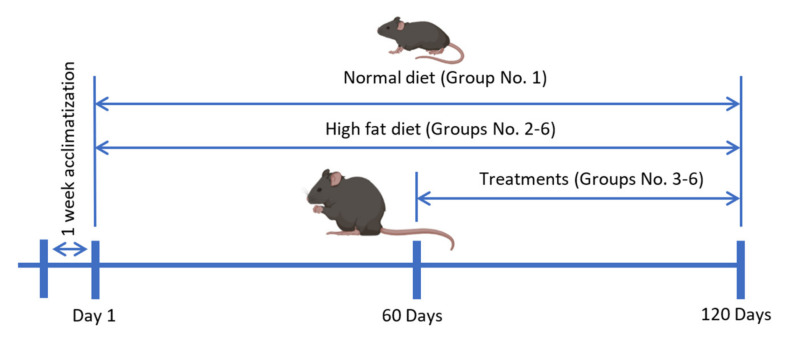
The timeline of the animal study.

The duration of the animal study was four months and the treatment was started at the end of the second month (Figure 13). The plant extract and compounds were dissolved in PBS. The administration was done daily by oral gavage based on their body weight, and the ND and HFD groups were administered the vehicle solvent. The body weight and food intake of the mice were monitored once a week. At the end of the treatment period, the mice were fasted overnight and then sacrificed under anaesthesia using ketamine (100 mg/kg) and xylazine (10 mg/kg). Blood samples were collected by cardiac puncture. Liver and total adipose tissue were collected for all animals, weighed and fixed in 10% buffered formalin.

#### 4.4.2. Biochemistry Analysis

The serum samples were separated by centrifugation at 10,000 rpm and 4 °C for 10 min, then sent to the biochemistry lab to measure liver enzymes (ALT, AST), triglycerides (TAG), total cholesterol (TC), high-density lipoprotein (HDL), and low-density lipoprotein (LDL) using a biochemical analyser (Hitachi 902 autoanalyzer, Tokyo, Japan). The second portion of serum was processed using the Mouse Expanded Magnetic Panel for Metabolic Hormone (Millipore, Merck, Rahway, NJ, USA, Cat. MMHE-44K) to detect IL-6, TNF-α, and insulin. Furthermore, serum adiponectin concentrations were measured using a mouse adiponectin ELISA kit (Bertin Pharma, formerly SPI Bio, Montigny-le-Bretonneux, France, Cat. A05187). All tests were performed according to the manufacturer’s protocols. In addition, serum lipid ratios (Equations (1)–(4)) [22,40,62], a homeostasis model assessment for insulin resistance (HOMA-IR) (Equation (5)) [63,64], and quantitative insulin sensitivity check index (QUICKI) (Equation (6)) were calculated [16].

(1) Castelli’s Risk Index I (CRI-I)=TCHDL

(2) Castelli’s Risk Index II (CRI-II)=LDLHDL

(3) Atherogenic Coefficient=TC−HDLHDL

(4) Atherogenic Index=log10TGHDL

(5) HOMA-IR=Insulinmmol/L×Glucosemmol/L22.5

(6) QUICKI=1log10InsulinmU/L×log10Glucosemg/dL

#### 4.4.3. Measurement of Antioxidant Enzymes Activity in Liver Tissue

To evaluate the antioxidant activity of *O. aristatus* against NAFLD, the enzyme activities of malondialdehyde (MDA) and superoxide dismutase (SOD) in liver tissue homogenate were measured. Liver tissues were homogenized in cold 1× PBS using a homogenizer and centrifuged (12,000 rpm at 4 °C) for 5 min. Lipid peroxidation was assessed using a colourimetric assay kit (Lipid Peroxidation, BioVision, Waltham, MA, USA, Cat. K739-100), and an EnzyChrom kit (BioAssay Systems, Hayward, CA, USA, Cat. ESOD-100) was used to determine SOD activity. All procedures were performed according to the manufacturer’s instructions.

#### 4.4.4. Histopathological Assessment

The other part of the liver tissue was harvested and fixed in 10% buffered formalin, placed in a cassette, and processed using a tissue processor (LeicaTP1020-1-1, Leica Biosystems, Nußloch, Baden-Wurttemberg, Germany) for 24 h. The tissues were then embedded in paraffin using an embedder (Leica EG 1160) and kept at 0 °C overnight. The paraffin-embedded blocks were sectioned (5 µm thick) using a rotary microtome (Leica RM2135, Leica Biosystems, Nußloch, Baden-Wurttemberg, Germany) and stained with hematoxylin and eosin (H&E, Baton Rouge, LA, USA) [65]. Briefly, the procedure involved deparaffinization in xylene and rehydration in decreasing concentrations of ethanol to distilled water, hematoxylin stain, differentiation, eosin, desiccation, clarification and assembly with Dibutylphthalate Polystyrene Xylene (DPX) Mountant (Merck, USA, Cat. 1.00579.0500). Tissue sections were examined under a light microscope (Leica DM750, Leica Bioystems, Nußloch, Baden-Wurttemberg, Germany) and scored by a pathologist. In addition, a Masson’s Trichrome stain (Sigma-Aldrich, USA, cat. HT15-1kt) was performed according to the manufacturer’s protocol. NAFLD development and the degree of recovery between groups were evaluated using the NAFLD activity score (NAS, Kuwait City, Kuwait) [66], and the degree of fibrosis was assessed according to a classification system commonly used in clinical practice (Table 5) [67].

### 4.5. In Vitro Study

#### 4.5.1. Cell Culture

HepG2 cells were obtained from ATCC (HB-8065-ATCC, USA). After thawing, cells were cultured in Eagle’s minimal essential medium (EMEM: ATCC, Cat. 30-2003) supplemented with 10% fetal bovine serum (FBS), non-essential amino acids 1× (SAFC, St. Louis, MO, USA, Cat. M7145), and penicillin-streptomycin solution (100 IU/mL and 100 µg/mL, respectively; ATCC, Cat. 30-2300). Growth was maintained under standard conditions at 37 °C in a humidified 5% CO_2_ atmosphere. At 80% confluency, cells were trypsinized (0.05% trypsin & 0.53 mM EDTA; Sigma-Aldrich, USA, Cat. T4049) for further experiment or subculture.

#### 4.5.2. Cytotoxicity Assay

Cytotoxicity was estimated using the CCK-8 assay (MedChem Express, Monmouth Junction, NJ, USA, Cat. HY-K0301). HepG2 cells were seeded at 4000 cells/100 uL/well in a 96-well plate overnight. After 12 h, different concentrations (i.e., 1000, 500, 250, 125, 62.5, 31.25, 15.6 µg/mL) of each fraction of *O. aristatus* were added. A fresh culture medium alone was used as a negative control. Each well was incubated at 37 °C with 10 µL CCK-8 solution for 2 h, and the read at an absorbance of 450 nm was made after 24 h, 48 h, and 72 h of starting the treatment. Cell toxicity percentage (CT%) was calculated using Equation (7) [68].

(7) CT%=CAbs−SAbsCAbs×100

where C_Abs_ is the mean of negative control absorbance value, and S_Abs_ is the mean of sample absorbance value.

#### 4.5.3. Induction of Steatosis in the Cells

After reaching 50% confluency, cells were exposed to a mixture of fatty acids 0.25 mM (1:2), i.e., palmitic acid ≥99% (Sigma-Aldrich, USA, Cat. P0500) and oleic acid 99% (Alfa Aesar, Lancashire, United Kingdom, Cat. ALF.031997.06) conjugated to bovine serum albumin (BSA) in a ratio of 1:5 for a further 24 h. For all the tested conditions, media were starved without FBS (serum-free medium) and supplemented with 1% BSA. Post-steatotic cells were exposed to the treatment for further 24 h [52].

#### 4.5.4. Detection of Intracellular Fat Accumulation

After treatment with fatty acids, the HepG2 cell medium was removed, gently washed with PBS and 100 µL of Nile red working solution (300 ng/mL Nile red (Sigma, USA, Cat. 19123). After that, 1 µg/mL Hoechst 33342 (Sigma, USA, Cat. 875756-97-1) in PBS was added to each well and incubated for 15 min at 37 °C and 5% CO_2_ in the dark. Then, cells were washed with PBS and imaged directly with a fluorescence microscope with excitation/emission maxima 488/550 nm for the Nile red and 350/461 nm for Hoechst 33342 [52,69,70]. The intracellular fat accumulation per cell was presented as a relative fold change as compared to the negative control (0.5 mM FA), which was computed in two steps. First, the Nile red (indicating intracellular fat droplets) readings were normalized to the reading of Hoechst 33342, which indicated the cell number using a standard curve. Second, the fold change of each reading was calculated compared to the negative control.

### 4.6. Enzyme Antioxidant Activities

#### 4.6.1. Protein Quantification

HepG2 cells were seeded into a T-25 flask at a density of 5 × 10^5^ cells. After 12 h, cells were exposed to the fatty acid mixture for 24 h, then treated for an additional 24 h, except for the negative control and 1% BSA flasks. Each flask was then washed twice with PBS, trypsinized and collected in a microcentrifuge tube and kept on ice. Then, the cells were lysed by adding 500 µL of ice-cold lysis buffer (0.1 M Tris/HCl, pH 7.4 containing 0.5% Triton X-100, 5 mM β-mercaptoethanol and 0.1 mg/mL PMSF). The Bradford assay was used to quantify the protein content as previously described and BSA was used as a calibration standard [71,72].

#### 4.6.2. Superoxide Anion Radical Scavenging Activity

The SOD Assay Kit-WST (Canvax Biotech, Córdoba Spain, Cat CA061) was used by following the kit’s instructions. Measurement was performed on 96-well microplates, and the absorbance was measured at 450 nm via a microplate reader. Reaction volumes were added as specified in the kit’s manual. Calculation of SOD activities (inhibition rate%) was conducted using the standard calibration curve, then normalised to the total protein content. All samples were surveyed in triplicates.

#### 4.6.3. Lipid Peroxidation Inhibition Assay

Lipid peroxidation inhibition assay was performed by the malondialdehyde (MDA) Colorimetric Assay Kit (Elabscience, Wuhan, China, Cat. E-BC-K025-S), which uses the thiobarbituric acid (TBA) method [73]. MDA in the catabolite of lipid peroxide reacts with TBA and the absorbance was measured at 532 nm after 30 min of incubation at room temperature using a spectrophotometer. The results were normalised to the total protein content.

### 4.7. Mitochondria Potential Membrane

The mitochondrial membrane potential (Δψ_m_) drives the generation of ATP by mitochondria, which was measured using the fluorescent probe Tetramethylrhodamine Methyl Ester (TMRM) as described previously with some modifications [74]. Briefly, HepG2 cells were seeded at 5 × 10^5^ density in a 96-well plate. After 24 h of the treatment, a fresh media with TMRM (100 nM) was added and incubated for 30 min in dark. The media was then gently aspirated and washed with 100 µL of 1X PBS and 0.2% BSA, and 10 µM carbonyl cyanide 3-chlorophenyl-hydrazone was used to collapse the mitochondrial membrane potential. The microplate was read on a fluorescence plate reader with an excitation/emission of 548/575 nm. The fluorescence values were corrected for the cell number by plotting a separate standard calibration curve of the Hoechst dye that correlated with the cell number [75].

### 4.8. Statistical Analysis

Statistical analysis and graphs were carried out using GraphPad Prism 8.4 (GraphPad Software, Inc., La Jolla, CA, USA). All the data were indicated as mean ± standard deviation (SD). The in vivo data were analysed using one-way ANOVA followed by Dunnett’s test or Mann–Whitney followed by Dunn’s test post hoc for parametric and non-parametric data, respectively. The in vitro data were analysed using two-way ANOVA followed by Tukey’s test post hoc. *p*-value < 0.05 was considered significant. The in vitro assays were separately performed in triplicate.

## 5. Conclusions

In the present work, the in vivo results obtained are supported by in vitro studies. Our results suggest that *O. aristatus* ethanolic extract improved NAFLD condition in mice by reducing hepatic steatosis, improving liver enzyme abnormalities, and protecting against atherosclerotic complications. More importantly, *O. aristatus* increases the liver’s antioxidant capacity and improves liver metabolism; thus, several related diseases including obesity and diabetes mellitus would also be alleviated. In addition, fractionation of the standardized extract showed that polar fractions have the most beneficial effects. Although the ethanolic fraction showed the best anti-NAFLD effect based on the parameters tested, the beneficial effect of the aqueous fraction is not negligible. Hence, this study underscores the usefulness of the communal consumption of the aqueous extract as a daily tea for a broad range and multi-systematic health benefits. The results presented here support the effective use of *O. aristatus,* particularly the polar fractions to alleviate metabolic disorders, including NAFLD and CVD.

## Figures and Tables

**Table 1:** HPLC chromatography analysis of the main makers of *O. aristatus*.

Marker	Peak Name	RT ^a^	UC ^b^	Concentration ^c^
**Rosmarinic acid**	RA	7.242	1,066,841	61.96 ± 0.009
**3′-hydroxy-5,6,7,4′-tetramethoxyflavone**	TMF	11.001	28,302	0.72 ± 0.005
**Sinensetin**	SIN	13.787	126,360	2.94 ± 0.014
**Eupatorin**	EUP	14.619	147,799	6.89 ± 0.031

^a^ Retention time (minutes); ^b^ The peak’s area under the curve; ^c^ µg/mg extract.

**Table 2:** Pearson correlation between the insulin (mIU/L) and glucose (mg/dL) levels among the tested mice groups.

	Normal Diet	High-Fat Diet	HFD + Orlistat (10 mg/kg)	HFD + *O. aristatus* (200 mg/kg)	HFD + *O. aristatus* (400 mg/kg)	HFD + Rosmarinic Acid (10 mg/kg)
**Pearson correlation**	−0.89 *	0.99 **	−0.99 ***	0.97 **	−0.81	0.92 *

* *p* < 0.05, ** *p* < 0.01, *** *p* ≤ 0.001.

**Table 3:** The insulin-resistant (HOMA-IR) and sensitivity (QUICKI) indexes.

Parameter	Normal Diet	High-Fat Diet	HFD + Orlistat (10 mg/kg)	HFD *+ O. aristatus* (200 mg/kg)	HFD *+ O. aristatus* (400 mg/kg)	HFD + Rosmarinic acid (10 mg/kg)
**HOMA-IR**	0.49 ± 0.132 ***	11.69 ± 3.247	4.26 ± 0.271	3.58 ± 1.188	1.70 ± 0.463 **	10.42 ± 3.215
**QUICKI**	0.438 ± 0.021 ***	0.274 ± 0.010	0.309 ± 0.003 **	0.319 ± 0.014 ***	0.355 ± 0.017 ***	0.278 ± 0.011

HOMA-IR: Homeostatic model assessment for insulin resistance. QUICKI: quantitative insulin-sensitivity check index. HOMA-IR, a value < 1.0: insulin-sensitive, >1.9: early insulin resistance, >2.9: significant insulin resistance [13]. HOMA-IR of 2.0 corresponded to normal liver fat [14], and a cut-off value of 4.5 was associated with NAFLD [15]. Lower QUICKI values indicate greater insulin resistance, a score below 0.382 ± 0.007, 0.331 ± 0.010, and 0.304 ± 0.007 are considered nonobese, obese and diabetic, respectively [16]. * *p* < 0.05, ** *p* < 0.01, *** *p* < 0.001 compared to the high-fat diet group. Values were presented as mean ± SD, *n* = 5.

**Table 4:** NAFLD activity scoring (NAS) of the liver tissues.

	Steatosis	Lobular Inflammation	Hepatocyte Ballooning	Total	Fibrosis
ND	0	0	0	0	0
HFD	2.6 ± 0.548	1	0	3.6 ± 0.548	0
HFD + Orlistat (10 mg/kg)	1.4 ± 1.142	1	0	2.4 ± 1.142	0
HFD + *O. aristatus* (200 mg/kg)	2.2 ± 1.304	0.8 ± 0.447	0	3.0 + 1.732	0
HFD + *O. aristatus* (400 mg/kg)	1.2 ± 1.304	0.6 ± 0.548	0	1.8 + 1.789	0
HFD + Rosmarinic acid (10 mg/kg)	2.8 ± 0.447	1	0	3.8 ± 0.447	0

The total NAS score represents the sum of scores for steatosis, lobular inflammation, and ballooning and ranges from 0–8. Total scores of 0–2 was considered not NASH; 3–4 was evenly divided among those considered not NASH, borderline, or positive for NASH; 5–8 considered NASH. The data were represented as mean ± SD, *n* = 5.

**Table 5:** NAFLD Activity Score (NAS) Components.

Item	Score	Extent	Definition and Comment
**Steatosis**	0	<5%	Refers to the amount of surface area involved by steatosis as evaluated on low to medium power examination.
	1	5–33%	
	2	>33–66%	
	3	>66%	
**Lobular Inflammation**	0	No foci	Acidophil bodies are not included in this assessment, nor is portal inflammation.
	1	<2 foci	
	2	2–4 foci	
	3	>4 foci	
**Hepatocyte Ballooning**	0	None	“Few” means rare but definite ballooned hepatocytes as well as diagnostically borderline cases.
	1	Few balloon cells	
	2	Many cells/prominent ballooning	
**Fibrosis**	0	No fibrosis	The main determinants of fibrosis are the degree of expansion of fibrotic areas between portal tracts.
	1	Mild: portal fibrosis without septa	
	2	Moderate: portal fibrosis with few septa	
	3	Severe: numerous septa without cirrhosis	
	4	cirrhosis	

## Data Availability

Data available within article.
